# Impact of cervical lymph node dissection on accessory spinal nerve XI function: Case series and literature review

**DOI:** 10.1016/j.ijscr.2025.112122

**Published:** 2025-10-28

**Authors:** Saout Arrih Badr, Bijou Walid, Oukessou Youssef, Rouadi Sami, Abada Reda, Mahtar Mohamed

**Affiliations:** Department of Otolaryngology, Head and Neck surgery, Ibn Rochd University Hospital, Faculty of Medicine and Pharmacy, Hassan II University, Casablanca, Morocco

**Keywords:** Case series, Accessory spinal nerve, Risk factors, Dissection, Cervical lymph nodes

## Abstract

**Introduction:**

Cervical lymph node dissection can damage the accessory spinal nerve, causing motor and pain disorders. This study evaluates the impact of these lesions and investigates the associated risk factors.

**Methods:**

Prospective study of 29 patients who underwent cervical lymph node dissection. Assessment of accessory spinal nerve function was performed by clinical examination and electromyogram on day 28 and 6 months post-operatively. The EMG parameters analyzed were onset latency and motor amplitude. Electrophysiological and clinical criteria were established to distinguish neurapraxia from axonotmesis: neurapraxia was defined by conduction block with normal distal latencies (<3 ms) and preserved amplitudes (>5 mV), while axonotmesis was characterized by prolonged latencies (≥3 ms) and reduced amplitudes (≤5 mV) with signs of axonal degeneration. Statistical analysis was performed using Mann-Whitney, Wilcoxon, and McNemar tests and linear regression (*p* < 0.05).

**Results:**

Damage to the accessory spinal nerve was common after cervical lymph node dissection: trapezius atrophy (72 %), scapular detachment (32 %), C2 hypoesthesia (34 %). EMG revealed a mean latency of 2.78 ms (34 % pathological) and a mean amplitude of 3.04 mV (84 % less than 5 mV). Malignant pathology significantly influenced EMG amplitude (*p* = 0.026). At 6 months, significant improvement was observed: recovery of joint amplitudes, reduction in muscle atrophy (*p* < 0.05), reduction in hypoesthesia to 5.1 %, and improvement in EMG latencies (2.6 % pathological vs. 30.8 % initially).

**Conclusion:**

Cervical lymph node dissection frequently causes damage to the accessory spinal nerve in the form of axonotmesis. Despite gradual clinical improvement (recovery of joint range of motion, reduction in muscle atrophy), electrophysiological abnormalities persist at 6 months, reflecting a prolonged recovery process requiring 12 to 18 months. Individual anatomical variations justify personalized management. This study highlights the importance of prolonged follow-up and appropriate preventive strategies to optimize functional recovery after dissection.

## Highlights

• Cervical lymph node dissection causes functional alterations of the accessory spinal nerve XI even when it is not severed, with axonotmesis-type lesions confirmed by electromyography.
• The study reveals high rates of complications: atrophy of the trapezius and sternocleidomastoid muscles, scapular detachment, and sensory disturbances.
• Malignant pathology significantly worsens functional impact, advanced age is correlated with sensory disorders, and certain anatomical variations (non-bifurcated nerve) predispose to complications.
• A statistically significant clinical improvement is observed at 6 months for strength, trophicity, and mobility, but recovery is not complete, highlighting the importance of specialized follow-up and surgical prevention.

## 1 Introduction

Performing a cervical lymph node dissection is not without risks, particularly regarding vascular and neural lesions. Anatomical structures of the neck, such as the accessory spinal nerve, the internal jugular vein, and the sternocleidomastoid muscle, are frequently exposed during this procedure. The accessory spinal nerve is often vulnerable due to its proximity to the targeted lymph node territories, especially those in the retrospinal region (IIb). The accessory spinal nerve plays a fundamental role in the innervation of the trapezius and sternocleidomastoid muscles. Its injury results in muscular weakness, limitation of shoulder movement amplitude, and chronic pain [1,2].

The clinical impact of these accessory spinal nerve lesions highlights the necessity to identify associated risk factors and to develop effective prevention and rehabilitation strategies. From this perspective, the objective of this study is to evaluate the impact of cervical lymph node dissection on the function of the accessory spinal nerve in the short and medium term and to identify risk factors associated with injuries to this nerve [3,4].

This case series has been reported in line with the PROCESS guidelines [7].

## 2 Materials and methods

This is a prospective study conducted on 29 patients who underwent cervical lymph node dissection between January 2025 and May 2025. To obtain a homogeneous series, we included all patients who underwent cervical lymph node dissection regardless of the initial indication, and who had a pathology report of the surgical specimen. Patients with a history of shoulder and upper limb pathology, patients who had previous cervical lymph node dissections, and patients who received adjuvant radiotherapy sessions before the initial evaluation of shoulder function were excluded from the series.

Data collection included age and sex, medical history, tumor stage, operated side, type of lymph node dissection performed, nerve thickness, nerve bifurcation, nerve path in relation to the internal jugular vein, nerve traction, resection of the sternocleidomastoid muscle (SCM), resection of the internal jugular vein (IJV), and pathological analysis of the collected lymph nodes, particularly those from territory IIb.

The evaluation of nerve function was performed starting from postoperative day 28 using a clinical examination including: Testing of the SCM and trapezius muscles, assessment of their trophicity, assessment of scapular mobility, measurement of shoulder joint amplitudes using a goniometer, and investigation of upper limb sensibility disorders. The clinical examination was completed with an electromyogram (EMG) to measure the motor nerve conduction of the accessory spinal nerve. The parameters studied included onset latency in milliseconds (ms) and the amplitude of the muscle action potential in millivolts (mV). Onset latencies are considered normal below 3 ms, while motor amplitudes are deemed adequate above 5 mV. Patients in our study were summoned from 6 months postoperatively. Of the 29 patients initially included, the objective was to measure their evolution by performing a second clinical examination, complemented by an electromyogram (EMG).

The Mann-Whitney test was used to evaluate the influence of different factors on the clinical picture and EMG results. The Wilcoxon signed-rank test and McNemar's test were used to follow the evolution of clinical and paraclinical parameters. Finally, linear regression analysis was used to examine factors influencing the evolution of EMG results. Statistical significance was defined as a *P* value <0.05.

This case series has been reported in line with the PROCESS Guideline [7].

## 3 Results

### 3.1 Study population characteristics

A total of 29 patients (50 operated sides) were included in this study. The cohort comprised 18 males (62.1 %) and 11 females (37.9 %) with a mean age of 58.3 years (range 25–79 years). The majority of patients (20 cases, 69.0 %) presented with malignant pathology, while 9 patients (31.0 %) had infectious conditions. Regarding the operated side, 21 cases involved unilateral dissection (12 right-sided, 9 left-sided) and 8 patients underwent bilateral procedures. The most common surgical procedure was lateral neck dissection including level IIb (46 sides, 92.0 %), while 3 sides (6.0 %) underwent triangular dissection, and 1 side (2.0 %) had posterolateral dissection (II-V). Table 1 presents the complete baseline demographic and clinical characteristics of the study population (Fig. 1, Fig. 2).

**Table 1:** Baseline demographic and clinical characteristics.

Characteristic	n (%)
Age (years), mean ± SD	58.3 ± 14.2
Sex	
Male	18 (62.1)
Female	11 (37.9)
Pathology	
Malignant	20 (69.0)
Infectious	9 (31.0)
Side of operation	
Right	12 (41.4)
Left	9 (31.0)
Bilateral	8 (27.6)
Type of surgery	
Lateral dissection	46 (92.0)
Triangular dissection	3 (6.0)
Posterolateral dissection	1 (2.0)

Fig. 2 **Distribution of abduction amplitudes.**

**Figure:**
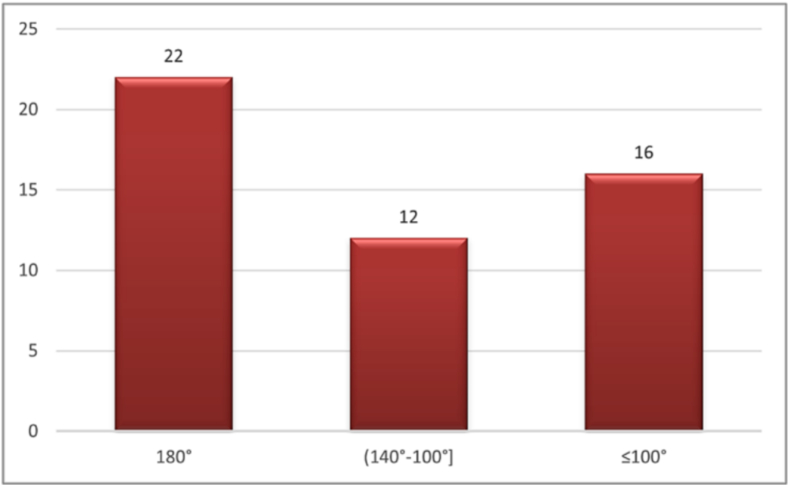

This figure illustrates the distribution of shoulder abduction measurements at day 28 post-operatively, demonstrating the variability in functional outcomes among patients.

Alt-text: Fig. 2

### 3.2 First postoperative evaluation

1. Joint amplitudes

2. Muscle strength

Fig. 1 **Distribution of anteflexion amplitudes.**

**Figure:**
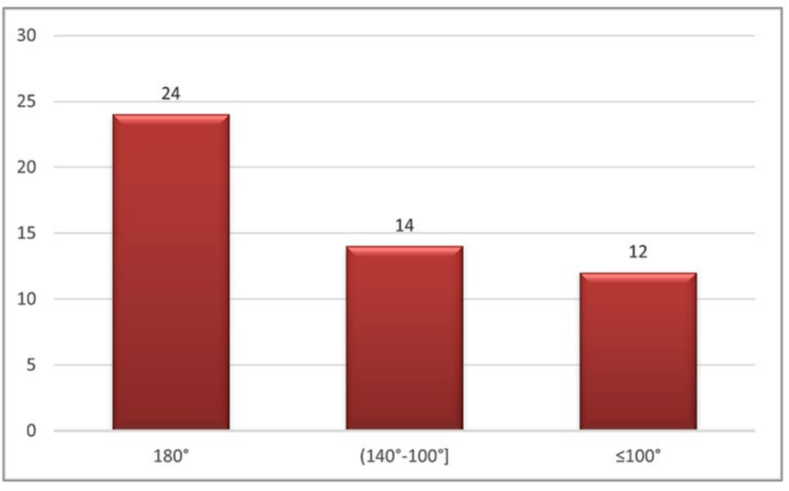

This figure displays the distribution of shoulder anteflexion measurements across all 50 operated sides at the first postoperative evaluation (day 28), showing the range and frequency of movement limitations.

Alt-text: Fig. 1

Fig. 3, Fig. 4, which demonstrate the distribution of muscle strength ratings on a scale from 1 to 5 for both muscle groups:

Fig. 3

**Figure:**
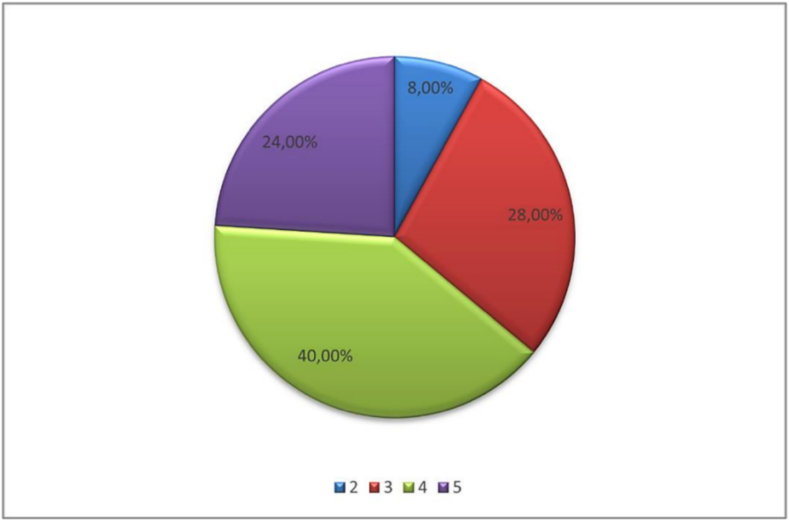

This figure shows the frequency distribution of strength ratings for the sternocleidomastoid muscle, with ratings ranging from 1 (complete weakness) to 5 (normal strength).

Alt-text: Fig. 3

3. Muscle trophicity and scapular winging

Fig. 4

**Figure:**
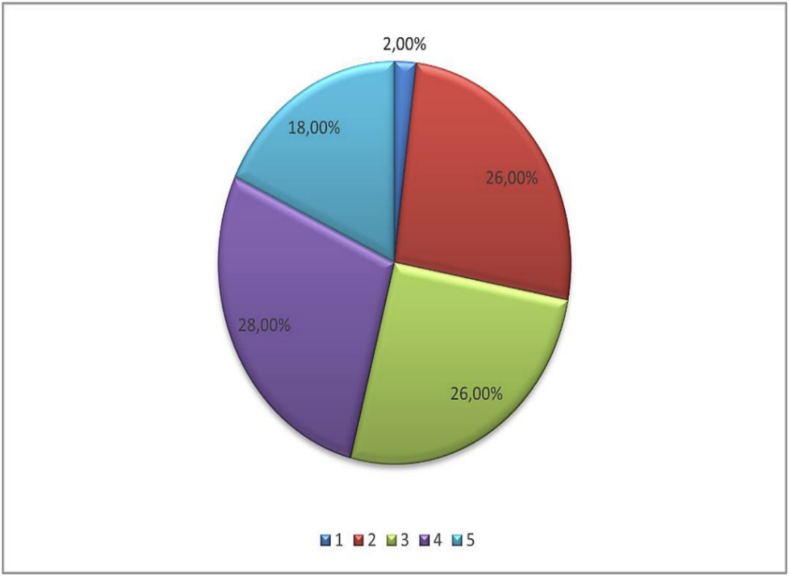

This figure presents the distribution of trapezius muscle strength assessments, illustrating the spectrum of functional impairment following cervical lymph node dissection.

Alt-text: Fig. 4

Of the 50 operated sides, 36 presented trapezius muscle atrophy, representing 72 % of the total. Regarding the sternocleidomastoid muscle, amyotrophy was observed in 15 cases out of 50, thus representing 30 % of the total. Among the 50 operated sides, scapular winging was observed in 16 cases, representing 32 %.

For sensibility disorders, hypoesthesia of the C2 territory was noted in 17 cases, representing 34 %, and hypoesthesia of the C3 and C4 territories in a single case, representing 2 % of the total.

4. Electromyographic data

For readers less familiar with neurophysiology, electromyographic (EMG) evaluation provides objective measurements of nerve function through two key parameters: onset latency (the time delay for nerve signal transmission, normally <3 ms) and compound muscle action potential amplitude (the strength of muscle response, normally >5 mV). Abnormal values indicate nerve damage, with prolonged latencies suggesting conduction problems and reduced amplitudes indicating axonal loss.

➢ Onset latencies

Out of a total of 50 evaluated sides, the mean observed latency was 2.78392 milliseconds (ms), with a standard deviation of 1.328667 ms. This latency time varied between a minimum of 0.926 ms and a maximum of 9.24 ms.

Percentiles provide a more detailed view of the distribution of latency times (Fig. 5). The 25th percentile was 2.02 ms, meaning that 25 % of the cases had a latency below this value. Similarly, the 75th percentile was 3.29 ms, indicating that 75 % of the cases had a latency below this limit. In our study, we defined a latency threshold at 3 ms. The results thus showed that 34 % of cases exceeded the threshold.

➢ Amplitudes of the compound muscle action potential

Fig. 5 **Analysis of action potential latencies.**

**Figure:**
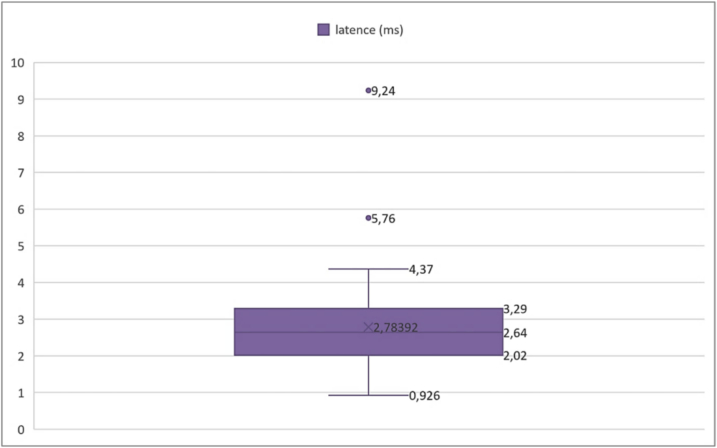

This box plot displays the distribution of onset latencies measured in milliseconds, showing quartiles, median values, and outliers across all 50 evaluated sides.

Alt-text: Fig. 5

The amplitudes of the evoked potentials were also measured. The results showed a mean amplitude of 3.04156 mV, with a standard deviation of 1.611716 mV. The minimum recorded was 0.34 mV, while the maximum was 6.4 mV. Analyzing the percentiles (Fig. 6), the 25th percentile was 1.5075 mV, meaning that 25 % of the amplitudes were below this value. The 75th percentile was 4.03 mV, indicating that 75 % of the amplitudes were below this threshold. For the amplitudes, we established a threshold of 5 mV. The results indicated that 84 % of cases presented rates below this threshold (Table 2).

Fig. 6 **Analysis of compound muscle action potential amplitudes.**

**Figure:**
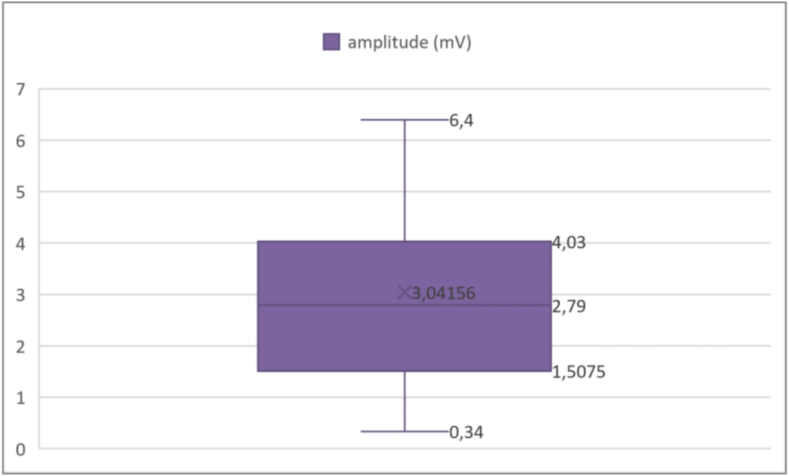

This figure presents the statistical distribution of muscle action potential amplitudes measured in millivolts, illustrating the extent of functional impairment across the study population.

Alt-text: Fig. 6

**Table 2:** Summary of key electrophysiological results.

Parameter	Mean ± SD	Range	Abnormal (n, %)
Onset latency (ms)	2.78 ± 1.33	0.93–9.24	17 (34 %)
Amplitude (mV)	3.04 ± 1.61	0.34–6.4	42 (84 %)

### 3.3 Relationship between clinical/surgical data and electromyographic (EMG) and clinical results

1. Relationship between demographic data and EMG:

Analysis of the relationship between patients' demographic data (age and sex) and electromyographic (EMG) results was performed using the Mann-Whitney test. The results showed that neither age (≤60 years vs >60 years) nor sex had a significant impact on EMG latencies and amplitudes (*p* > 0.05).

2. Impact of initial pathology on EMG:

The sample was divided into two main groups according to the initial pathology, malignant and infectious. The Mann-Whitney test was used to analyze the impact of these pathologies on electromyographic (EMG) results.

The results showed that, for latencies, the *p*-value was greater than 0.05. However, for amplitudes, the p-value was less than 0.05 (*P* = 0.026), indicating that patients with malignant pathology had a more marked decrease in amplitude (Fig. 7).

3. Impact of dissection types and intraoperative data on EMG:

Fig. 7 **Amplitudes: comparison between patients with malignant (blue) and infectious (orange) pathologies. (For interpretation of the references to colour in this figure legend, the reader is referred to the web version of this article.)**

**Figure:**
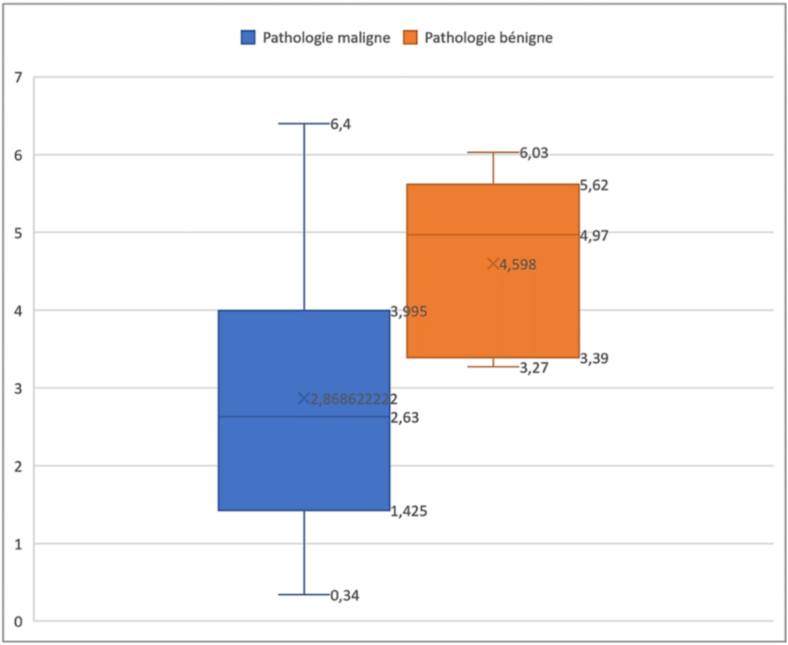

This comparative chart displays amplitude measurements in millivolts for malignant versus infectious pathology groups, demonstrating the statistical difference between the two conditions.

Alt-text: Fig. 7

Two main types of dissection were performed in our sample, lateral dissection and triangular dissection. All these dissections included the retrospinal area IIb. Statistical analysis did not find any significant difference, with *p*-values greater than 0.05 for both measurements. While statistical significance was not achieved, it is important to note the observed proportional changes: lateral dissection showed a mean amplitude reduction of 45 % compared to normal values, while triangular dissection demonstrated a 52 % reduction. These proportional differences, though not statistically significant in our small sample, may have clinical relevance and warrant consideration for type II error due to limited sample size. Additionally, a posterolateral dissection (II-V) was performed in a single case. This specific dissection presented a high latency of 4.1 ms and a reduced amplitude of 1.11 mV. Given that the dissection of area V represented a unique observation in our sample, it is difficult to formulate generalizable conclusions from these results.

The intraoperative characteristics of the accessory spinal nerve examined included the nerve diameter (thin or thick), the type of nerve (single or bifurcated), and its position relative to the internal jugular vein (anterior or posterior). However, the evaluation of the impact of nerve traction was limited by the presence of a single case. The test results showed that for each of the compared pairs, the *p*-values were greater than 0.05.

4. Distribution of EMG results according to pathological analysis of retrospinal lymph nodes IIb

A Mann-Whitney analysis was performed to compare the latencies and amplitudes of the accessory spinal nerve between cases where the retrospinal lymph nodes IIb were metastatic and those where they were not. The results showed that the *p*-values were greater than 0.05 for both measurements.

5. Analysis of clinical results based on demographic, pathological, and dissection types

The Mann-Whitney test was also used to explore factors influencing the clinical picture (joint amplitudes, muscle strength, muscle trophicity with scapular mobility). The results showed that only one correlation was found between the presence of a single, non-bifurcated accessory spinal nerve and the atrophy of the corresponding SCM muscle (*P* = 0.012). Furthermore, a significant correlation was also observed between the presence of postoperative hypoesthesia and the patients' age (*p* < 0.05), suggesting that older patients were more likely to develop this complication.

### 3.4 Differential analysis of postoperative results from both evaluations

1. Joint amplitudes

During the second measurement, an overall improvement was observed. For anteflexion, a total of 30 cases presented a maximum anteflexion of 180° and a single case with an amplitude of 60°. The other measured amplitudes varied between 110° and 165°. For abduction, 29 cases had an abduction amplitude of 180°, while two cases presented amplitudes of 90° and 50°, respectively. The other 8 amplitudes varied between 140° and 160°.

To evaluate the statistical significance of this difference, the Wilcoxon signed-rank test for paired samples was used. The test result showed a *p*-value less than 0.05. We can conclude that the difference observed between the two measurements was statistically significant, reflecting a real clinical progression.

2. Muscle strengths

During the second measurement for the trapezius muscle, the median increased to 4, and the 75th percentile also increased to 5, showing an improvement in muscle performance for a majority of participants.

During the second measurement for the SCM muscle, the median remained at 4, the 75th percentile at 5, indicating a general stability in the distribution of SCM muscle strength among participants. However, the 25th percentile increased to 4, suggesting that at least 75 % of cases had a muscle strength greater than or equal to 4. (Fig. 8, Fig. 9 display the comparative analysis of muscle strength between the two evaluation periods).

3. Muscle trophicity and scapular winging (Fig. 10, Fig. 11, Fig. 12)

Fig. 8 **Comparative analysis of trapezius muscle strength.**

**Figure:**
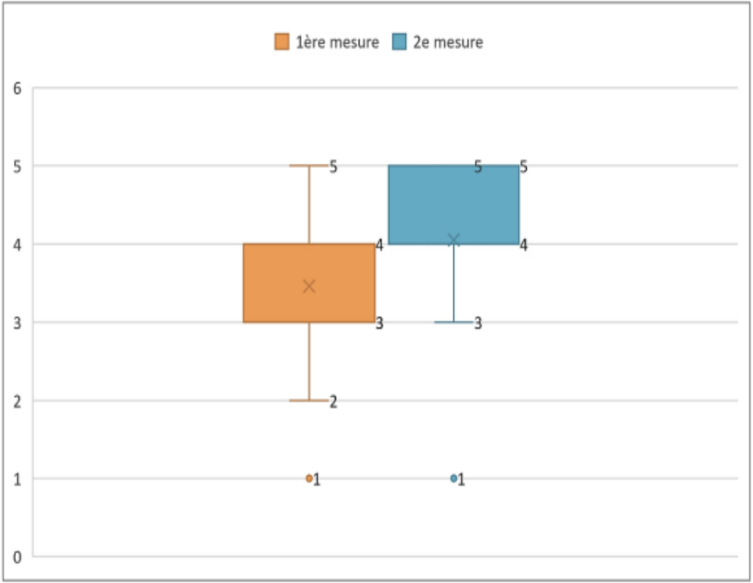

This figure presents side-by-side comparison of trapezius muscle strength ratings at day 28 and 6 months post-operatively, showing the evolution of functional recovery.

Alt-text: Fig. 8

Fig. 9 **Comparative analysis of SCM muscle strength.**

**Figure:**
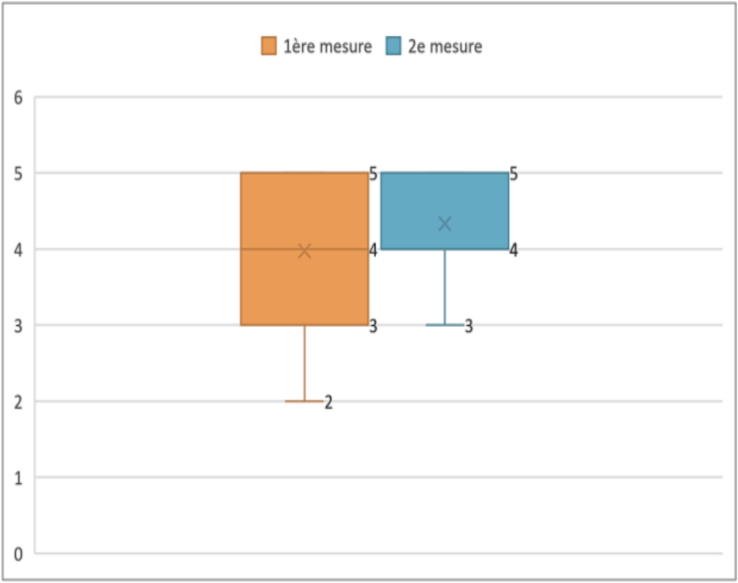

This figure illustrates the comparative distribution of sternocleidomastoid muscle strength between the first and second evaluations, demonstrating stability in strength recovery patterns.

Alt-text: Fig. 9

The evolution of muscle trophicity and scapular mobility is presented in the following analysis, with accompanying figures showing the proportional changes over time:

Fig. 10 **Evolution of trapezius muscle trophicity.**

**Figure:**
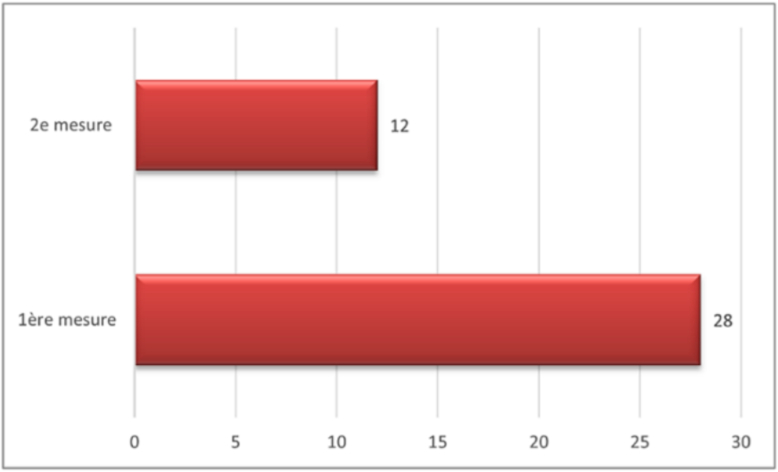

This figure demonstrates the change in trapezius muscle atrophy rates between day 28 and 6 months, showing the number and percentage of cases with muscle trophicity improvement over time.

Alt-text: Fig. 10

In our sample, a *p*-value less than 0.05 indicated a significant difference between the two evaluations.

Fig. 11 **Evolution of SCM muscle trophicity.**

**Figure:**
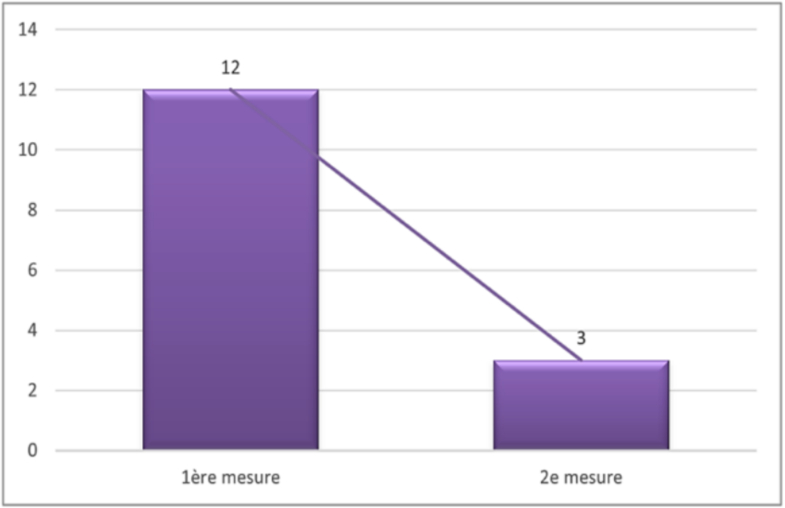

This figure presents the progression of sternocleidomastoid muscle atrophy, displaying both the absolute numbers and percentages of cases showing improvement in muscle trophicity.

Alt-text: Fig. 11

The analysis showed a *p*-value less than 0.05, suggesting that the reduction in the proportion of SCM amyotrophy was significant.

Fig. 12 **Evolution of scapular mobility.**

**Figure:**
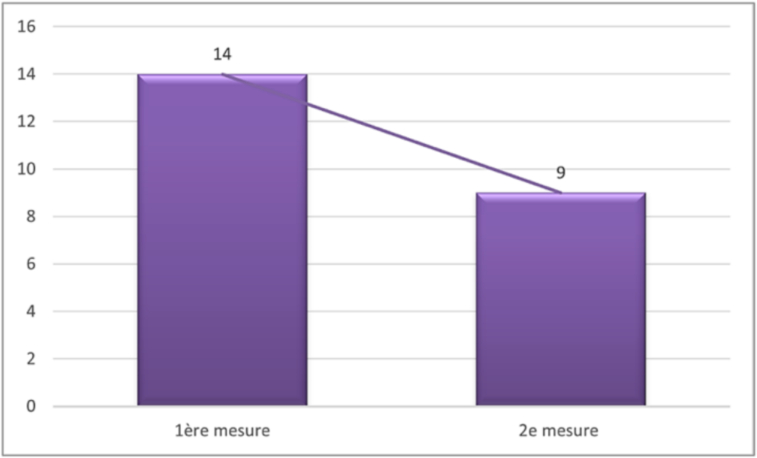

This figure illustrates the changes in scapular winging frequency between the two evaluation periods, showing both improved and persistent cases.

Alt-text: Fig. 12

The obtained *p*-value was 0.13. This means that the observed difference in the proportions of scapular winging between the two measurements was not significant, indicating the persistence of this complication.

4. Sensibility disorders

During the second evaluation, only 2 sides (5.1 %) still presented hypoesthesia, all located at the C2 dermatome level. The p-value obtained through McNemar's test was less than 0.05, suggesting an overall improvement in our patients.

5. Electromyographic Evolution (Fig. 13, Fig. 14)

Fig. 13 **Variation of onset latencies in EMG.**

**Figure:**
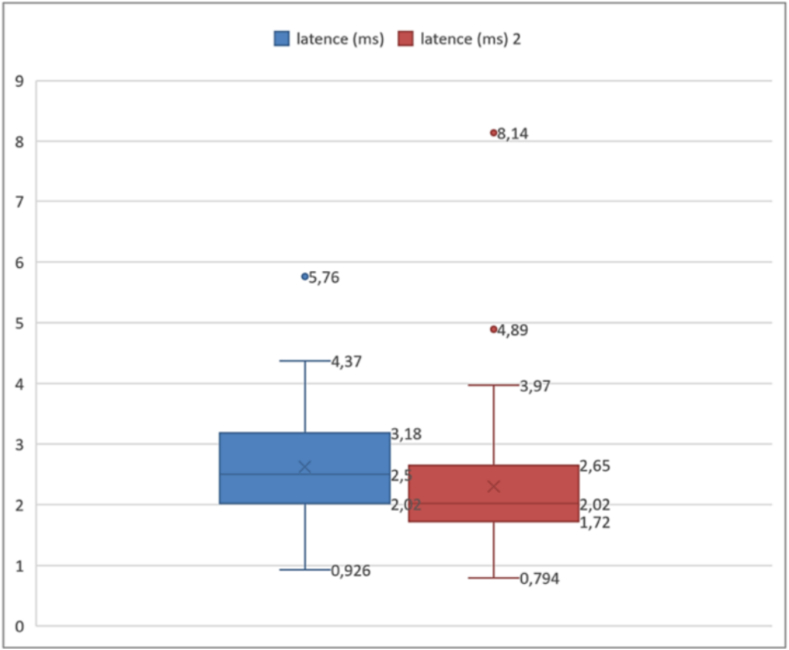

This figure displays box plots comparing onset latency measurements at day 28 versus 6 months, showing median values, quartiles, and the distribution of recovery patterns.

Alt-text: Fig. 13

Comparison of quartiles showed that the interquartile range was wider at 1 month (from 2.02 to 3.18 ms) than at 6 months (from 1.72 to 2.65 ms). This indicates a greater variability of latencies initially, which decreased at 6 months, suggesting a more uniform recovery of subjects over time. Thus, comparing with the defined threshold of 3 ms, 30.8 % of cases initially presented a higher latency. At 6 months post-operative, this percentage decreased to 2.56 %. The Wilcoxon test for paired samples showed a *p*-value less than 0.05, indicating a significant increase in onset latencies of the action potential between one- and six-months post-operative.

Fig. 14 **Evolution of compound muscle action potential amplitudes.**

**Figure:**
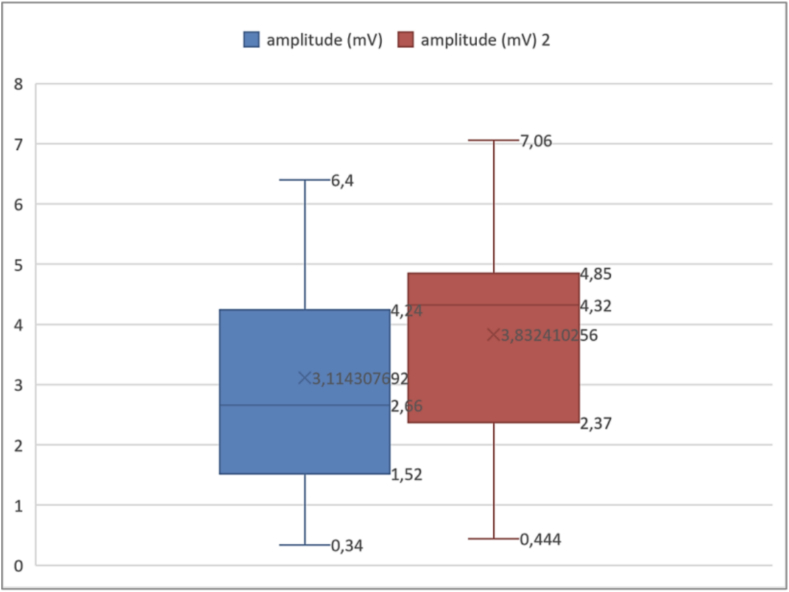

This figure shows the comparative distribution of amplitude measurements between day 28 and 6 months, illustrating the median values, quartile ranges, and overall recovery trends.

Alt-text: Fig. 14

The median amplitude was approximately 2.66 mV initially and higher, at approximately 4.32 mV at 6 months. Comparison of quartiles shows that the interquartile range was wider at 1 month (from 1.52 to 4.24 mV) than at 6 months (from 2.37 to 4.85 mV). This suggests a more uniform recovery of subjects. The *p*-value obtained from the Wilcoxon test for paired samples was less than 0.05. While statistical significance was achieved, it is important to note that the rate of cases with an amplitude less than 5 mV went from 82.1 % to 79.5 %, showing a modest proportional improvement of 2.6 % despite the statistical significance. This nevertheless remained below the predefined threshold in the majority of cases, highlighting the persistent nature of electrophysiological abnormalities despite clinical improvement.

### 3.5 Impact of clinical/surgical factors on the evolution of EMG results

The analyzed parameters included demographic data (age, sex), surgical data (types of dissection, characteristics of the accessory spinal nerve), pathological data (infectious or tumoral), and histological data (retrospinal lymph nodes IIb). We used multiple linear regression to evaluate their impact on EMG latencies and amplitudes. No factor showed significant influence. The *p*-values were all greater than 0.05. The absence of statistically significant associations should be interpreted cautiously, as our limited sample size may have resulted in type II error, potentially masking clinically relevant relationships between these factors and EMG outcomes.

## 4 Discussion

### 4.1 High frequency and severity of accessory spinal nerve injury

The most frequent neurological complication related to selective lymph node dissection remains the impairment of the accessory spinal nerve. The study by Chiesa-Estomba et al. demonstrated that 10 % of dissections had an impact on shoulder motor function. During the dissection of lymph nodes in territory IIb, intraoperative traction, dissection, and electrocoagulation of the nerve can cause neuropraxia or axonotmesis. Our study confirms that even when the nerve is anatomically preserved, functional damage occurs in the majority of cases, with 72 % of patients developing trapezius muscle atrophy and 84 % showing abnormal EMG amplitudes. These lesions manifest as pain during upper limb mobilization, limitation of abduction, scapular winging, and muscle atrophy, particularly of the trapezius muscle. These manifestations may differ from one patient to another, due to variations in muscle innervation. This is mainly provided by the XIth cranial nerve, but the motor roots of the cervical plexus also contribute [[1], [2], [3]].

### 4.2 Impact of surgical extent and pathology type

Lima et al.'s study aimed to identify the impact of dissection on the function of the accessory spinal nerve in two distinct groups. The first had undergone dissection including the retrospinal lymph node area IIb, and the other had undergone combined dissection of levels IIb and V. The results showed that 90 % of cases having undergone dissection of area V lymph nodes had upper limb abduction less than 90°. In comparison, only 56 % of cases with manipulation limited to level IIb presented similar restrictions. These results corroborate ours, where 32 % of cases had abduction less than 100°. This suggests that the extension of dissection to level V more significantly impacted the motor function of the shoulder [4]. Furthermore, our findings demonstrate that malignant pathology significantly worsens EMG outcomes compared to infectious conditions (*p* = 0.026), likely due to the pro-inflammatory environment and cytokine-mediated nerve damage associated with malignancy.

### 4.3 Electrophysiological evidence of axonotmesis and recovery patterns

Our electromyographic findings provide objective evidence that the majority of nerve injuries represent axonotmesis rather than simple neuropraxia. Giordano et al. compared two groups having undergone selective lateral dissections: group A, with retrospinal IIb dissection, and group B, with conservation of retrospinal lymph nodes. At 3 weeks post-operative, the amplitude of the evoked potential was 1.03 mV for group A versus 6.43 mV for group B, indicating better recovery in the group with preservation of the retrospinal area. The decrease in amplitude, observed after 21 days, was classified as axonotmesis, suggesting that less invasive surgery reduced morbidity and improved quality of life [5].

Our study confirms this pattern, with mean amplitudes of 3.04 mV at one month and improvement to 4.32 mV at six months, though still remaining below normal thresholds in most cases. This persistent electrophysiological abnormality despite clinical improvement suggests ongoing axonal regeneration that typically requires 12–18 months for completion.

### 4.4 Anatomical variations and age-related factors

Our analysis revealed important correlations between anatomical variations and clinical outcomes. We established a correlation between the presence of a non-bifurcated accessory spinal nerve and sternocleidomastoid muscle atrophy. The study by Shiozaki et al. emphasizes that an injury to the accessory nerve, when it is non-bifurcated, can occur because it can be confused with the greater auricular nerve or the transverse cervical nerve, thus increasing the risk of intraoperative injury [6].

Additionally, we found a significant correlation between advanced age (>60 years) and sensory disorders, which can be explained by age-related reduction in epidermal nerve endings and altered nociceptive perception, as previously documented in neuroanatomical studies.

### 4.5 Clinical implications and management recommendations

Based on our findings, several clinical implications emerge for surgical practice and postoperative management. Malignant pathologies, particularly head and neck cancers, were associated with significantly worse electrophysiological outcomes, requiring more intensive postoperative monitoring and rehabilitation. The surgical procedures most frequently associated with nerve complications in our cohort were lateral neck dissections including level IIb (92 % of cases), suggesting that particular attention should be paid to nerve preservation techniques in this region.

Regarding management recommendations based on nerve damage severity: patients with mild axonotmesis (latencies 3–5 ms, amplitudes 2–5 mV) showed good recovery potential with conservative management and physiotherapy; those with severe axonotmesis (latencies >5 ms, amplitudes <2 mV) required prolonged rehabilitation programs lasting 12–18 months; and patients with anatomical nerve variations (non-bifurcated nerves) needed individualized surgical approaches to minimize complications.

An important observation from our study concerns the differences in recovery timelines between clinical assessments and electrophysiological findings. While patients showed significant clinical improvement in joint mobility, muscle strength, and sensory function by 6 months, electrophysiological abnormalities persisted in the majority of cases (79.5 % still had amplitudes <5 mV). This discrepancy highlights the importance of prolonged electrophysiological follow-up beyond 6 months, as clinical improvement may mask ongoing nerve regeneration processes that require 12–18 months for completion.

## 5 Conclusion

Our prospective study highlights the significant impact of cervical lymph node dissection on shoulder function, even in the absence of anatomical lesion of the accessory spinal nerve. The functional alterations, corroborated by electromyogram, reveal a neurological impairment of the axonotmesis type, mainly related to tumor pathology. Anatomical variations also influence the clinical presentation of nerve injuries, justifying an individualized consideration during management. Despite a clinical improvement in the medium term, electrophysiological abnormalities persist, reflecting a prolonged recovery process.

## 6 Limitations

Several limitations must be acknowledged in this study. First, the relatively small sample size (29 patients, 50 operated sides) may have limited our statistical power to detect significant associations between various factors and outcomes. This small sample size increases the risk of type II error, particularly in comparisons where no statistically significant differences were found. Second, the short follow-up duration of 6 months may limit our ability to draw long-term conclusions about nerve recovery, as axonotmesis typically requires 12–18 months for complete regeneration. Third, the single-center design of our study may affect the generalizability of our findings to other institutions with different surgical techniques or patient populations. These limitations should be considered when interpreting our results and planning future multi-center studies with larger sample sizes and longer follow-up periods.

## CRediT authorship contribution statement

Saout Arrih Badr: Study concept and writing the paper

Bijou Walid: Study concept and correction of the paper

Oukessou Youssef: Study concept and correction of the paper

Rouadi Sami: Study concept and correction of the paper

Abada Reda: Study concept and correction of the paper

Mahtar Mohamed: Study concept and correction of the paper

## Consent

Written informed consent was obtained from the patient for publication of this case report and accompanying images. A copy of the written consent is available for review by the Editor-in-Chief of this journal on request.

## Ethical approval

Ethical approval has been exempted by our institution Chu ibn Rochd due to minimal risk and retrospective nature of the study.

## Provenance and peer review

Not commissioned, externally peer-reviewed.

## Sources of funding

None.

## Research registration

N/a

## Declaration of conflicts of interest

The authors declare having no conflicts of interest for this article.
